# BRIDGING THE GAP: SERVICE COORDINATION IN HUD HOUSING DURING COVID-19

**DOI:** 10.1093/geroni/igac059.1333

**Published:** 2022-12-20

**Authors:** Samara Scheckler, Jennifer Molinsky

**Affiliations:** Harvard University, Cambridge, Massachusetts, United States; Harvard University, Cambridge, Massachusetts, United States

## Abstract

Older HUD residents were vulnerable to COVID-19 due to their age, health, income, race, ethnicity, and resources. In combination, these factors increased risk to health and housing stability. While many HUD properties employ a service coordinator, this role is not universally adopted and impacts of these services are not well understood. This research surveyed service coordinators in mid-2020 and late-2021 to characterize older adult experiences living in public housing during the pandemic and ways service coordinators helped manage disruptions. Surveys were disseminated to 3,500 service coordinators. Findings identified needs in transportation, personal care, sociality, mental and physical healthcare, and food. Interventions included resource procurement, benefits management, technology access improvement, and linking residents to services. Tools included information dissemination, needs assessments, and partnership development. This work offers insight into the role of a service coordinator to mitigate health and housing risk. It becomes increasingly salient as HUD residents trend demographically older.

